# Configural but Not Featural Face Information Is Associated With Automatic Processing

**DOI:** 10.3389/fnhum.2022.884823

**Published:** 2022-04-13

**Authors:** Hailing Wang, Enguang Chen, JingJing Li, Fanglin Ji, Yujing Lian, Shimin Fu

**Affiliations:** ^1^School of Psychology, Shandong Normal University, Jinan, China; ^2^Department of Psychology, School of Social Sciences, Tsinghua University, Beijing, China; ^3^Department of Psychology and Center for Brain and Cognitive Sciences, School of Education, Guangzhou University, Guangzhou, China

**Keywords:** face, configural, featural, visual mismatch negativity (vMMN), automatic processing

## Abstract

Configural face processing precedes featural face processing under the face-attended condition, but their temporal sequence in the absence of attention is unclear. The present study investigated this issue by recording visual mismatch negativity (vMMN), which indicates the automatic processing of visual information under unattended conditions. Participants performed a central cross size change detection task, in which random sequences of faces were presented peripherally, in an oddball paradigm. In Experiment 1, configural and featural faces (deviant stimuli) were presented infrequently among original faces (standard stimuli). In Experiment 2, configural faces were presented infrequently among featural faces, or vice versa. The occipital-temporal vMMN emerged in the 200–360 ms latency range for configural, but not featural, face information. More specifically, configural face information elicited a substantial vMMN component in the 200–360 ms range in Experiment 1. This result was replicated in the 320–360 ms range in Experiment 2, especially in the right hemisphere. These results suggest that configural, but not featural, face information is associated with automatic processing and provides new electrophysiological evidence for the different mechanisms underlying configural and featural face processing under unattended conditions.

## Introduction

Faces are the indispensable visual stimuli for social interactions. It is well known that individual face perception relies on configural/global (i.e., second-order relations, the distance between facial features) and featural/local processing (i.e., the shape or size of the single feature), which represent distinct types of face processing associated with structural encoding units based on the functional model of facial processing (Bruce and Young, 1986; Maurer et al., 2002; Renzi et al., 2013). An increasing number of electrophysiological studies have shown that configural face processing precedes featural face processing, which supports the coarse-to-fine sequence of facial processing (Gao and Bentin, 2011; Goffaux et al., 2011; Peters et al., 2018).

Consistent with the above idea, a recent event-related potential (ERP) study using a face gender discrimination task observed that faces with low and high spatial frequency elicited the largest P1 and N170 amplitudes, respectively, among all conditions (Jeantet et al., 2019), where configural and featural face processing are believed to occur in low and high spatial frequency ranges, respectively. Furthermore, the time course of configural and featural face processing were investigated using different attentional paradigms (Wang et al., 2015, 2016, 2020; Wang and Fu, 2018). Participants were required to match faces based on configural and featural information. Configural and featural face processing elicited larger P1 and P2 components in the occipito-temporal cortex, respectively, under the face-attended condition. Taken together, these results suggested that configural and featural face processing follow a coarse-to-fine sequence.

However, the above studies all concentrated on the attended condition. There is evidence that faces can be processed even when they are presented without a particular focus of attention (Palermo and Rhodes, 2007). That is, the human brain is able to process faces automatically and assign them high priority. The visual mismatch negativity (vMMN) component has been described as a promising index for evaluating the automatic processing of stimulus changes. This component is usually examined via a passive oddball paradigm, in which frequent (standard) stimuli and infrequent (deviant) stimuli are presented at random. The potential difference between the deviant and standard stimuli is the vMMN, which is usually elicited over posterior scalp sites in the 200–400 latency range (Kimura et al., 2012; Csizmadia et al., 2021).

Notably, to obtain a reliable vMMN, it is necessary to ensure that the evoking stimuli are presented outside of the focus of attention (Stefanics et al., 2014). Previous studies used various methods to reduce attentional effects and investigated the automatic processing of aspects of facial stimuli, such as age, emotional expression and gender (Kimura et al., 2012; Csizmadia et al., 2021). Zhao and Li (2006) used a location discrimination task with an acoustic tone, to avoid contamination by attention to emotional facial stimuli, which were presented in the center of the visual field. The vMMN was observed in relation to sad and happy faces over occipital-temporal scalp sites in the 110–430 ms latency range. However, others have argued that auditory tasks might not be sufficient to guarantee that the attention is directed away from foveally presented stimuli, especially for salient emotional facial stimuli. As an alternative, they used a size-change detection task with a central fixation cross, and presented four different emotion facial stimuli peripherally (Stefanics et al., 2012; Csukly et al., 2013). The vMMN in the occipital-temporal area was obtained in early (150–220 ms) and later (250–360 ms) latency ranges.

As mentioned above, vMMN studies related to facial stimuli have focused on automatic processing of the social information provided by faces. To our knowledge, few studies have investigated unattended processing and encoding of structural facial information, which is essential for recognizing individuals. Recently, Wang et al. (2014) recorded the vMMN (140–320 ms) elicited by rotated and upright faces, and found that rotated faces had a larger vMMN relative to upright faces in the right occipito-temporal cortex. This result suggested that individuals are more sensitive to configural face processing even in the absence of attention, due to impairment of the configural face processing caused by rotation.

To address the question of whether face perception followed the coarse-to-fine sequence under the unattended condition, in the present study we investigated the vMMNs elicited by configural and featural face processing, respectively. Importantly, compared with face inversion, configural and featural face stimuli allowed us to independently manipulate the two levels of structural encoding of faces. To ensure that attention did not involve the processing of the eliciting stimuli, we asked the participants to detect infrequent changes in a central fixation cross, while standard and deviant face stimuli were presented peripherally (Stefanics et al., 2012; Kuldkepp et al., 2013; Chen et al., 2020). Additionally, previous studies suggested that the vMMN related to face stimuli in the higher latency range reliably reflects the automatic processing of faces, where the lower latency range might be confounded by changes of the face-sensitive N170 component (Astikainen and Heitanen, 2009). We expect to observe automatic processing of facial structural information in the occipito-temporal cortex in the later latency range. That is, deviant stimuli are expected to evoke more negative amplitudes than standard stimuli. Moreover, we hypothesize that configural face processing will elicit a larger and/or earlier vMMN than facial feature processing in the later latency range, if face processing occurs in a coarse-to-fine manner during automatic processing.

## Experiment 1

### Materials and Methods

#### Participants

A power analysis conducted with G*Power software (Faul et al., 2007) revealed that a total sample of 31 participants was required for a three-level (configural deviant vs. featural deviant vs. original standard faces) within-subjects ANOVA to detect medium effect sizes (*η2* p = 0.05) with 80% power, a 5% probability for type I error and a correction for non-sphericity of *e* = 1. In total, 36 students (18 females; age range: 19–26 years; mean age: 20.6 ± 1.9 years) were recruited from Tsinghua University. All participants were healthy and right-handed, and had normal or corrected-to-normal vision. The research protocol was approved by the Institutional Review Board (IRB) of the Department of Psychology, Tsinghua University. Written informed consent was obtained from each participant prior to the experiment.

#### Stimuli and Apparatus

**FIGURE 1:**
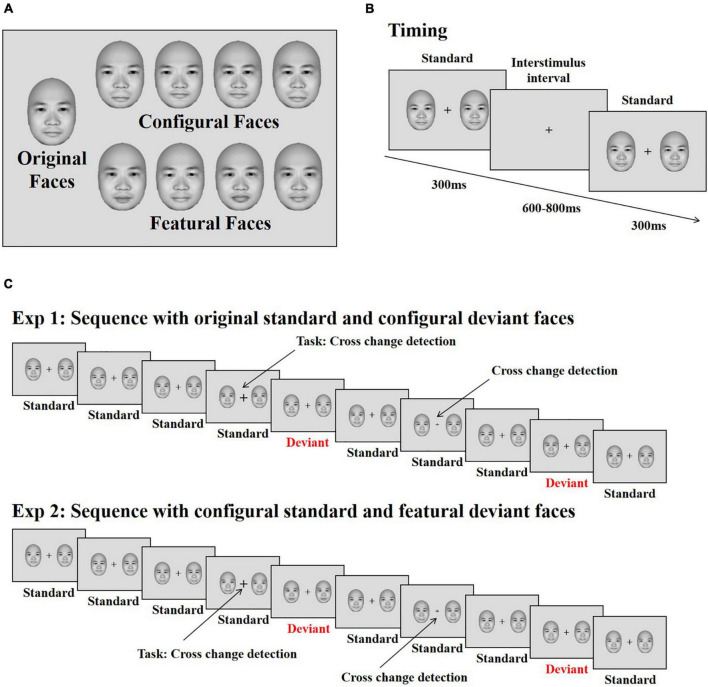
Stimuli and illustration of the experiment. **(A)** The original face and its modifications differing for changes in configural (the distance between eyes and between mouth and nose) and featural (the shape of the eye and mouth) information. **(B)** The presenting time of each stimulus. **(C)** The stimuli sequence applied in the experiments. Participants’ task was to detect the changes of the cross. The face stimuli were used from the FaceGen Modeller 3.5 (Toronto, ON, Canada).

The face stimuli were frontal view photos generated by FaceGen Modeller 3.5 (Toronto, ON, Canada). Based on the original face stimuli, configural and featural face stimuli were constructed with the same method used in previous studies (eight stimuli for each; Wang et al., 2015, 2016, 2020; Wang and Fu, 2018). Configural faces were constructed by manipulating the distance between the eyes, and between the mouth and nose. Featural face refers to the fact that the original faces’ eyes and mouth were replaced by other eyes and mouths (Figure 1A). All stimuli were presented on a 17-in. ViewSonic monitor (resolution: 1024 × 768; refresh rate: 100 Hz) using E-Prime 2.0 software (Psychology Software Tools, Inc., Sharpsburg, PA, United States) at a viewing distance of 60 cm. The stimulus size was 4° × 5.5° (113 × 156 pixels).

#### Procedure

Each trial started with the presentation of a black fixation cross for 200 ms, immediately followed by two faces (both of which were original, configural or featural faces) presented on both sides of the fixation cross for 300 ms against a gray background (RGB: 220, 220, 220); this was followed by an inter-stimulus interval (offset to onset of the next trial) of 600–800 ms (Figure 1B). The eccentricity of the two faces (measured as the distance between the center of each face and the central fixation cross) was 3.8°. Within each block, original faces served as standard stimuli, and configural and featural faces as deviant stimuli. Participants completed two blocks, each consisting of 480 trials. In total, 70% were standard trials, consisting of the original faces along with the standard cross mark (size of the cross was set to “24”), while 10% of the trials were target trials, presenting the original faces along with the target cross mark (size of the cross = “32” or “16”); the remaining 20% of trials were divided evenly between trials presenting deviant configural or featural faces, with the standard cross mark used for both sets. Participants were asked to ignore the peripheral stimuli and press “F” or “J” on the keyboard with the left or right index finger on detecting a change in size of the fixation cross (smaller or bigger, Figure 1C). The response keys were counterbalanced across participants.

#### Electroencephalogram (EEG) Recording and Analysis

Brain electrical activity was recorded from 64 scalp sites using Ag/AgCl electrodes mounted on an elastic cap (NeuroScan, Charlotte, NC, United States), and the physical reference electrode was located between CPz and Cz. Horizontal electrooculographies (EOGs) were recorded from two electrode sites at the outer canthi of each eye. Vertical EOGs were recorded from electrodes situated on the infraorbital and supraorbital regions of the left eye. The inter-electrode impedance was maintained below 5 kΩ throughout the electroencephalogram (EEG) recording session. The EEG and EOG readings were collected using a band-pass filter of 0.05–100 Hz and sampled at a rate of 500 Hz.

The electrophysiological data were analyzed using the Letswave toolbox freeware (Delorme and Makeig, 2004) in the Matlab environment (2017a, Natick, MA, United States). The EEG analyzing window was between −100 and 600 ms, and the 100 ms pre-stimulus EEG served as a baseline. The EEG data were band-pass filtered within the range of 0.1–30 Hz and re-referenced to the average of all electrodes. Ocular artifacts were removed by applying independent component analysis (ICA). Forty components were examined as potential artifacts, and one or two components were removed for each participant. The number of trials was 86 ± 12.6, 86 ± 12.6, and 603 ± 84.3 for configural deviant, featural deviant and original standard faces, respectively.

Based on previous face-related vMMN studies (Stefanics et al., 2012; Kecskés-Kovács et al., 2013; Kreegipuu et al., 2013) and the current potential distributions, the regions of interests were restricted to P7/8, PO7/8, and O1/2, which showed larger negativities for deviant relative to standard stimuli in the 200–440 ms range. The mean amplitudes in this range were used in vMMN-related analysis, which was tested by a three-way ANOVA [Stimuli (Original Standard vs. Configural Deviant vs. Featural Deviant faces) × Hemisphere (Left vs. Right) × Electrode (P7/P8 vs. PO7/PO8 vs. O1/O2)]. Peak amplitude (from baseline to peak) and peak latency values of the P1 (80–150 ms) and N170 (130–200 ms) components were analyzed using similar ANOVAs. Consistent with our previous studies (Wang et al., 2015, 2016, 2020; Wang and Fu, 2018), N2 and P3a were analyzed to eliminate contamination of the ERP results by stimuli novelty. The mean amplitude was analyzed for N2 (220–260 ms) from electrodes F1/2, FC1/2, and C1/2, and was tested by two-way ANOVA [Stimuli (Original Standard vs. Configural Deviant vs. Featural Deviant faces) × Hemisphere (Left vs. Right)]. The mean amplitude was analyzed for P3a (300–400 ms) from electrodes Fz, FCz, Cz, and CPz by one-way ANOVA [Stimuli (Original Standard vs. Configural Deviant vs. Featural Deviant faces)]. When necessary, Greenhouse-Geisser correction of the degrees of freedom was applied. Effect sizes were represented by partial eta-squared. In addition, Bonferroni correction was applied for multiple comparisons as a *post hoc* analysis (alpha level = 0.05).

### Results and Discussion

#### Behavioral Data

The hit rate, i.e., rate of successful detection of size changes in the target stimuli, was 89.80 ± 8.64%. The mean reaction time was 599 ± 67 ms.

#### Event-Related Potential Data

Regarding the P1 amplitude and latency, we observed no significant hemispheric differences. However, the right hemisphere had a larger and longer N170 than the left hemisphere [amplitude: −2.82 vs. −2.19 μV, *F*(1,35) = 4.99, *p* < 0.032, η*2 p* = 0.13; latency: 174 vs. 170 ms, *F*(1,35) = 4.39, *p* < 0.043, η*2 p* = 0.11].

Regarding the N2 and P3a components, we observed no significant differences in configural or featural face processing, due to a lack of a significant main effect of Stimuli [N2: *F*(2,70) = 2.623, *p* = 0.097; P3a: *F*(2,70) = 0.770, *p* = 0.442]. The interaction between Stimuli and Hemisphere was significant for N2 [*F*(2,70) = 3.880, *p* < 0.040, η*2 p* = 0.100]. *Post hoc* analysis showed that the original standard stimuli had a more negative amplitude than configural deviant stimuli in the right hemisphere (−0.96 vs. −0.71 μV, *p* < 0.038).

**FIGURE 2:**
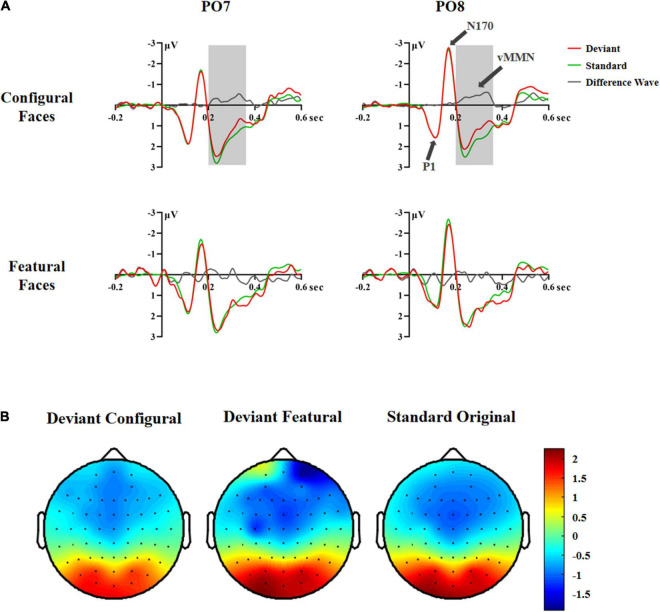
ERP responses in Experiment 1. **(A)** ERP responses to deviant and standard stimuli and deviant-minus-standard differential waveforms (vMMN, 200–360 ms). **(B)** Topographic maps of deviant and standard stimuli (200–360 ms).

A significant difference between deviant and standard stimuli was observed within the 200–440 ms time range based on the *F*-test [*F*(2,70) = 4.25, *p* < 0.030, η*2 p* = 0.11; configural deviant 1.03 vs. featural deviant 1.26 vs. original standard faces 1.28 μV], indicating the presence of vMMN (Figure 2). To investigate the time course of vMMN, we conducted separate ANOVAs across consecutive 40 ms latency windows within the 200–440 ms latency range (Wang et al., 2014). The results were as follows.

In the 200–240 ms latency range, the main effects of Hemisphere and Stimuli were significant [Hemisphere: *F*(1,35) = 6.81, *p* < 0.013, η*2 p* = 0.16; Stimuli: *F*(2,70) = 4.04, *p* < 0.033, *η2* p = 0.10]. *Post hoc* analysis showed that the right hemisphere had a more negative response than the left hemisphere (1.19 vs. 1.80 μV). The configural deviant faces elicited more negative responses than the original standard faces (1.34 vs. 1.60 μV, *p* < 0.029), and there was no difference between the featural deviant and original standard faces (1.56 vs. 1.60 μV), indicating a vMMN for configural but not featural faces.

In the 240–280 ms latency range, the main effect of Stimuli was significant, [*F*(2,70) = 5.25, *p* < 0.011, η*2 p* = 0.13]. *Post hoc* analysis showed that the configural deviant faces elicited more negative responses than the original standard faces (1.51 vs. 1.81 μV, *p* < 0.022), and there was no difference between the featural deviant and original standard faces (1.81 vs. 1.81 μV).

In the 280–320 ms latency range, the main effect of Stimuli was significant, [*F*(2,70) = 7.20, *p* < 0.005, η*2 p* = 0.17]. *Post hoc* analysis showed that the configural deviant faces elicited more negative responses than the original standard faces (0.92 vs. 1.30 μV, *p* < 0.002), and there was no difference between the featural deviant and original standard faces (1.24 vs. 1.30 μV).

In the 320–360 ms latency range, the main effect of Stimuli was significant, [*F*(2,70) = 7.20, *p* < 0.005, η*2 p* = 0.17]. *Post hoc* analysis showed that the configural deviant faces elicited more negative responses than the original standard faces (0.68 vs. 1.11 μV, *p* < 0.004), and there was no difference between the featural deviant and original standard faces (1.01 vs. 1.11 μV).

In the 360–400 and 400–440 ms latency ranges, no significant effects were found (*Fs* ≥ 0.873, *ps* ≥ 0.399), indicating that there was no vMMN both for configural and featural faces.

Taken together, the above vMMN results showed that configural, but not featural, face information elicited a vMMN component in the 200–360 ms latency range, indicating that configural rather than featural face information is subject to automatic processing. However, according to previous studies (Stefanics et al., 2012; Wang et al., 2014), presenting the same stimuli as both deviants and standards allowed us to investigate the vMMN by comparing physically identical stimuli, and reduced the influence of physical differences between stimuli on vMMN. Therefore, in experiment 2 we compared the ERP waveforms to physically identical facial stimuli using a deviant-standard-reverse oddball paradigm (i.e., configural deviant vs. featural standard, featural deviant vs. configural standard).

## Experiment 2

### Materials and Methods

#### Participants

A power analysis conducted with G*Power software (Faul et al., 2007) revealed that a total of 28 participants was required for a 2 (deviant vs. standard stimuli) × 2 (configural vs. featural face) within-subjects ANOVA to detect medium effect sizes (η*2 p* = 0.05) with 80% power, a 5% probability for type I error and a correction for non-sphericity of *e* = 1. Thirty students (19 females; age range: 18–25 years; mean age: 20.2 ± 2.2 years) were recruited from Tsinghua University. All participants were healthy and right-handed, and had normal or corrected-to-normal vision. The research protocol was approved by the Local Institutional Review Board (IRB) of the Department of Psychology, Tsinghua University. Written informed consent was obtained from each participant prior to the experiment.

#### Stimuli, Procedure, Electroencephalogram Recording and Data Analysis

The stimuli, equipment, procedure and analysis were similar to those in Experiment 1, but there were some differences, as follows. Configural faces were presented as standard stimuli and featural faces were the deviant stimuli in one experimental block; the standard and deviant face stimuli were swapped for the other experimental block. Each block consisted of 480 trials, 70% of which used standard stimuli along with the standard cross mark (size of the cross = “24”); 10% of the trials were target trials, in which the standard stimuli were presented along with the target cross mark (size of the cross = “32” or “16”); the remaining 20% of trials used deviant stimuli and the standard cross mark, as did the featural faces. The number of trials was 86 ± 14.2, 90 ± 7.7, 316 ± 27.7, and 304 ± 49.7 for configural deviant, featural deviant, configural standard and featural standard faces, respectively. For vMMN-related analysis, four-way ANOVA was used [Stimuli (Standard vs. Deviant) × Face (Configural vs. Featural) × Hemisphere (Left vs. Right) × Electrode (P7/P8 vs. PO7/PO8 vs. O1/O2)]. The P1 and N170 components were analyzed. N2 was tested by three-way ANOVA [Stimuli (Standard vs. Deviant) × Face (Configural vs. Featural) × Hemisphere (Left vs. Right)]. P3a was tested by two-way analysis [Stimuli (Standard vs. Deviant) × Face (Configural vs. Featural)].

### Results and Discussion

#### Behavioral Data

A *t*-test showed no significant difference between the configural and featural standard face blocks in hit rate (*t*_29_ = 1.143, *p* = 0.264, 92.79% ± 6.43% vs. 91.19% ± 6.85%) or reaction time (*t*_29_ = −0.932, *p* = 0.360, 580 ± 69 ms vs. 584 ± 73 ms).

#### Event-Related Potential Data

Regarding the P1 amplitude and latency, we observed no significant group differences. However, the right hemisphere had a larger N170 than the left hemisphere [−3.07 vs. −2.36 μV; *F*(1,29) = 7.18, *p* < 0.012, η*2 p* = 0.20]. This effect was modulated by Face, as there was a significant interaction between Hemisphere and Face [*F*(1,29) = 4.26, *p* < 0.048, η*2 p* = 0.13]. The follow-up analysis showed that the hemisphere effect was larger for configural (left −2.33 vs. right hemisphere −3.11 μV, *p* < 0.006) than featural (left −2.40 vs. right hemisphere −3.03 μV, *p* < 0.027) face processing. No significant effects were found for N170 latency.

Regarding the N2 and P3a components, we observed no significant differences in configural or featural face processing, due to the lack of a significant main effect of Face [N2: *F*(1,29) = 0.05, *p* = 0.823; P3a: *F*(1,29) = 1.674, *p* = 0.206]. The interaction effect of Face × Stimuli × Hemisphere was significant for N2 [*F*(1,29) = 4.46, *p* < 0.043, η*2 p* = 0.133]. *Post hoc* analysis showed that the featural standard stimuli had a more negative amplitude than the featural deviant stimuli in the left hemisphere (−0.89 vs. −0.62 μV, *p* < 0.016).

A significant difference between deviant and standard stimuli was obtained within the 200–440 ms time range based on the *F*-test [*F*(1,29) = 4.32, *p* < 0.047, η*2 p* = 0.13; deviant 1.16 vs. standard stimuli 1.29 μV]. Similar to Experiment 1, we conducted separate ANOVAs across consecutive 40 ms latency windows within the 200–440 ms latency range to investigate the time course of vMMN. The results were as follows.

In the 200–240 ms latency range, the main effect of Hemisphere was significant [*F*(1,29) = 5.97, *p* < 0.021, η*2 p* = 0.17], indicating that the right hemisphere had a more negative response than the left hemisphere (1.00 vs. 1.48 μV). The interaction effect of Stimuli × Electrode × Hemisphere was significant [*F*(2,58) = 4.65, *p* < 0.015, η*2 p* = 0.14]. *Post hoc* analysis revealed a hemisphere effect for deviant stimuli at P7/8 (left 0.57 vs. right 0.01 μV, *p* < 0.022) and PO7/8 (left 1.77 vs. right 1.09 μV, *p* < 0.008), as seen for standard stimuli at PO7/8 (left 1.80 vs. right 1.20 μV, *p* < 0.014).

In the 240–280 ms latency range, the significant interaction effect of Stimuli × Electrode × Hemisphere [*F*(2,58) = 5.43, *p* < 0.008, η*2 p* = 0.16] was due to the fact that the deviant stimuli had more negative responses than the standard stimuli at P8 (0.95 vs. 1.18 μV, *p* < 0.010) and PO8 (1.77 vs. 2.02 μV, *p* < 0.014), indicating that there was a vMMN component in the right occipital-temporal cortex.

In the 280–320 ms latency range, the main effects of Stimuli and Face were significant [Stimuli: *F*(1,29) = 6.94, *p* < 0.013, η*2 p* = 0.19; Face: *F*(1,29) = 10.60, *p* < 0.003, η*2 p* = 0.27]. *Post hoc* analysis showed that the deviant stimuli elicited more negative responses than the standard stimuli (1.29 vs. 1.50 μV), and that configural faces had more negative responses than featural faces (1.29 vs. 1.50 μV).

**FIGURE 3:**
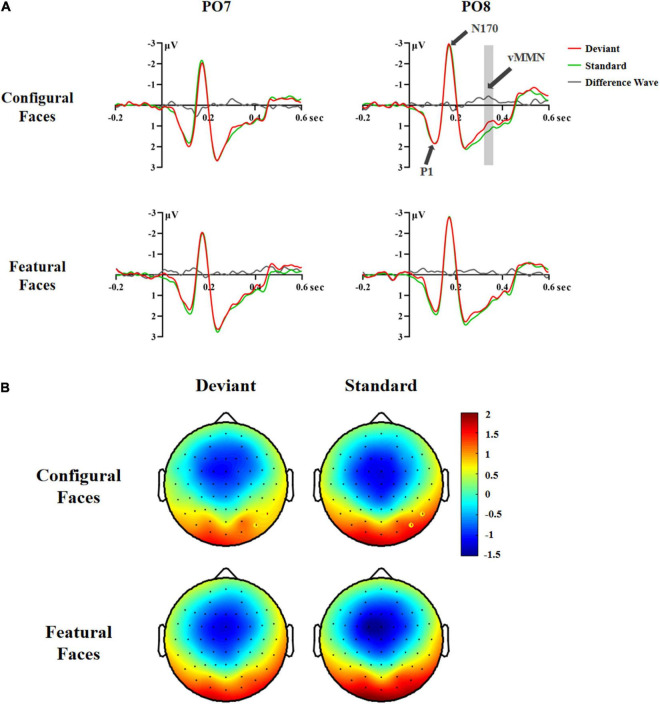
ERP responses in Experiment 2. **(A)** ERP responses to deviant and standard stimuli and deviant-minus-standard differential waveforms (vMMN, 320–360 ms). **(B)** Topographic maps of deviant and standard stimuli for configural and featural faces (320–360 ms). P8 and PO8 electrodes are marked with circles.

In the 320–360 ms latency range, the main effects of Stimuli and Face were significant [Stimuli: *F*(1,29) = 5.42, *p* < 0.027, η*2 p* = 0.16; Face: *F*(1,29) = 8.82, *p* < 0.006, η*2 p* = 0.23]. *Post hoc* analysis showed that the deviant stimuli elicited more negative responses than the standard stimuli (1.07 vs. 1.25 μV), and that configural faces had more negative responses than featural faces (1.06 vs. 1.26 μV). The interaction effect of Stimuli × Electrode × Hemisphere was significant [*F*(2,58) = 3.72, *p* < 0.037, η*2 p* = 0.11]. Follow-up analysis showed that the deviant stimuli had more negative responses than the standard stimuli at P8 (0.87 vs. 1.09 μV, *p* < 0.005) and PO8 (1.09 vs. 1.38 μV, *p* < 0.006). More importantly, the interaction effect of Stimuli × Face × Hemisphere was significant [*F*(1,29) = 7.02, *p* < 0.013, η*2 p* = 0.20]. *Post hoc* analysis showed that the deviant configural faces elicited more negative responses than standard configural faces in the right hemisphere (0.85 vs. 1.23 μV, *p* < 0.016). This effect was modulated by Electrode, as the effect interaction of Stimuli × Face × Electrode × Hemisphere was significant [*F*(2,58) = 4.83, *p* < 0.013, η*2 p* = 0.14]. Follow-up analysis showed that configural deviant faces had more negative responses than standard configural faces at P8 (0.67 vs. 1.08 μV, *p* < 0.002) and PO8 (0.78 vs. 1.31 μV, *p* < 0.005), indicating the emergence of the vMMN for configural face processing in the right occipital-temporal cortex (Figure 3).

In the 360–400 and 400–440 ms latency ranges, no significant effects were found (*Fs* ≥ 3.687, *ps* ≥ 0.065), indicating that there was no vMMN for configural or featural faces.

Taken together, the above vMMN results showed that face stimuli elicited a vMMN component in the 240–360 ms latency range. More importantly, configural, but not featural, face processing elicited a vMMN component in the right occipital-temporal cortex (P8/PO8) in the 320–360 ms latency range, indicating that configural rather than featural face information tends to be processed automatically.

### General Discussion

How is facial structural information processed in the absence of attention? In the current study, we investigated this issue by evaluating the vMMN component elicited by configural and featural face information. Configural information refers to the spatial relations among facial components, whereas featural information pertains to the shape or size of each facial component. A visual detection task involving the center of the visual field was employed, while standard and deviant face stimuli appeared in the periphery. As expected, vMMN emerged during the later latency range (200–360 ms) in both experiments. No electrophysiological evidence was found for featural face processing in any time window, whereas configural face processing elicited a reliable vMMN during later latency ranges (Experiment 1: 200–360 ms, Experiment 2: 320–360 ms), especially on the right hemisphere.

Face stimuli encompass not only various types of social information, but also different forms of structural information, which contribute to the recognition of individual faces and processing of social information contained in faces (Fallshore and Bartholow, 2003; PrKachin, 2003; Zhao and Bentin, 2011; Wang et al., 2020). Previous studies demonstrated that vMMN was sensitive to social category information conveyed by faces (Kovács-Bálint et al., 2014; Kovarski et al., 2017; Zhang et al., 2018). Furthermore, we observed that face structural information was able to elicit vMMN when configural and featural deviant faces were presented. To our knowledge, few studies have investigated the automatic face structural information encoding processes by independently manipulating configural and featural face processing. The current findings suggested that face structural information encoding can occur pre-attentively.

Regarding the specific aim of the current study, our data showed that configural face information was more easily processed automatically than featural face information. Using different face perception and category discrimination tasks, previous studies observed that configural and featural face processing elicited larger P1 and N170/P2 components, respectively (Wang et al., 2015, 2016, 2020; Wang and Fu, 2018; Jeantet et al., 2019), suggesting a coarse-to-fine sequence in face processing. In the present study, however, only configural deviant faces elicited robust negative amplitudes compared to standard face stimuli, when the face stimuli were unrelated to the ongoing task. One possible reason for this is related to the neural mechanisms of the visual system. It is well known that spatial (e.g., shape, color) and temporal (e.g., motion, duration) information are processed in the ventral (what) and dorsal (where) visual pathways, respectively. Previous studies suggested that stimuli violating temporal regularities appeared earlier than spatially deviant stimuli (for a review, see Kremláček et al., 2016). The dorsal visual pathway was confirmed to contribute to configural face processing, but not to featural face processing (Zachariou et al., 2017). This is probably due to the involvement of the dorsal visual pathway; configural face information were extracted easily relative to featural face information, even when the face stimuli were unattended.

Why did configural but not featural face processing elicit a substantial vMMN? The variation in distance between the facial features during configural face processing appeared somewhat atypical, whereas featural face processing appeared typical and unexceptional. It is plausible that the salience or novelty of configural face processing was greater compared to featural face processing, such that configural deviant stimuli more readily violated the regularity of the oddball paradigm; however, we ruled out this possibility. The frontocentral N2 and P3a components are considered indicators of attentional orientation to novel and significant events in the environment (Polich, 2007; Folstein and Van Petten, 2008; Lawson et al., 2012). In this study, they did not differ between configural and featural face processing under the attended condition, although configural face processing is more easily recognized than featural face processing (Wang et al., 2015, 2020; Wang and Fu, 2018). This was also the case under the unattended condition of the present study. Thus, our finding cannot be attributed to salience differences between configural and featural face processing. Our results were consistent with previous studies, which observed MMN only in the context of global auditory processing of local patterns (List et al., 2007). At the same time, the vMMN elicited by inverted faces was attenuated relative to upright ones (Susac et al., 2004; Chang et al., 2010; Kimura et al., 2012), because face inversion impeded configural face processing (Yin, 1969; Bartlett and Searcy, 1993). Taken together, these findings demonstrated the different mechanisms underlying automatic processing of configural and featural face information.

Previous studies also showed that a difference between deviant and standard stimuli arose in the early latency range (∼70–220 ms; Stefanics et al., 2012; Wang et al., 2014). However, we failed to observe differences between deviant and standard stimuli for the P1 and N170 components. In this study, P1 and N170 were insensitive to configural and featural face processing; we observed no significant differences between configural and featural face processing for these components. These results seem to be inconsistent with previous studies, which found that configural and featural face processing were dissociated for P1 and N170 under the attended condition (Wang and Fu, 2018; Jeantet et al., 2019; Wang et al., 2020). However, it should be noted that attention conditions differed across studies. The present data indicated that the early ERP components cannot reflect automatic processes, and were insensitive to face structural encoding information under the unattended condition.

We also found that the face-sensitive N170 was larger in the right than left hemisphere. It should be noted that, in Experiment 2, we observed a right hemispheric advantage for configural face processing for N170 and vMMN (320–360 ms). This laterality was also observed in other studies (Rossion et al., 2000; Scott and Nelson, 2006; Maurer et al., 2007; Renzi et al., 2013; Cattaneo et al., 2014; Wang and Fu, 2018). Compared with previous reports, our data suggested right hemisphere dominance for configural face processing even when face stimuli were unattended. Despite the fact that we used the same stimuli and task in Experiments 1 and 2, we did not observe lateralization in Experiment 1, and the latency range of vMMN was longer in Experiment 1 than Experiment 2, which might be related with the stimuli sequence. In contrast to Experiment 2, the standard stimuli come from the original faces in Experiment 1, which might make the infrequent configural faces more salient and result in earlier and longer vMMN latency than featural faces. But our N2 and P3a results did not find the significant difference between configural and featural faces. Further studies are needed to determine the reason for this.

In conclusion, the vMMN component was observed in both experiments during later latency ranges, showing that vMMN is sensitive to facial structural information. More importantly, compared with featural face information, configural face information elicited a substantial vMMN over a relative wide latency range, suggesting that the processing of configural rather than featural face information can occur independent of attention. These data provided new evidence of different underlying mechanisms of configural and featural face processing, from the perspective of automatic processing under unattended conditions.

## Data Availability Statement

The raw data supporting the conclusions of this article will be made available by the authors, without undue reservation.

## Ethics Statement

The studies involving human participants were reviewed and approved by the Local Institutional Review Board (IRB) of the Department of Psychology, Tsinghua University. The patients/participants provided their written informed consent to participate in this study.

## Author Contributions

HW and SF designed the research. HW and FJ obtained the data. HW, EC, JL, and YL analyzed the data. HW, EC, JL, and SF wrote and edited the manuscript. All authors contributed to the article and approved the submitted version.

## Conflict of Interest

The authors declare that the research was conducted in the absence of any commercial or financial relationships that could be construed as a potential conflict of interest.

## Publisher’s Note

All claims expressed in this article are solely those of the authors and do not necessarily represent those of their affiliated organizations, or those of the publisher, the editors and the reviewers. Any product that may be evaluated in this article, or claim that may be made by its manufacturer, is not guaranteed or endorsed by the publisher.
